# A FinnGen pilot clinical recall study for Alzheimer’s disease

**DOI:** 10.1038/s41598-023-39835-7

**Published:** 2023-08-03

**Authors:** Valtteri Julkunen, Claudia Schwarz, Juho Kalapudas, Merja Hallikainen, Aino-Kaisa Piironen, Arto Mannermaa, Hanna Kujala, Timo Laitinen, Veli-Matti Kosma, Teemu I. Paajanen, Reetta Kälviäinen, Mikko Hiltunen, Sanna-Kaisa Herukka, Sari Kärkkäinen, Tarja Kokkola, Mia Urjansson, FinnGen, Hilkka Soininen, Sami Heikkinen, Tomi P. Mäkelä, Anastasia Kytölä, Andrea Ganna, Anu Jalanko, Aoxing Liu, Arto Lehisto, Awaisa Ghazal, Elina Kilpeläinen, Elisabeth Widen, Elmo Saarentaus, Esa Pitkänen, Hanna Ollila, Hannele Laivuori, Henrike Heyne, Huei-Yi Shen, Joel Rämö, Juha Karjalainen, Juha Mehtonen, Jyrki Pitkänen, Kalle Pärn, Katja Kivinen, Elisa L. Lahtela, Mari E. Niemi, Mari Kaunisto, Mart Kals, Mary Pat Reeve, Mervi Aavikko, Nina Mars, Oluwaseun Alexander Dada, Pietro Della Briotta Parolo, Priit Palta, Rigbe Weldatsadik, Risto Kajanne, Rodos Rodosthenous, Samuli Ripatti, Sanni Ruotsalainen, Satu Strausz, Shabbeer Hassan, Shanmukha Sampath Padmanabhuni, Shuang Luo, Susanna Lemmelä, Taru Tukiainen, Timo P. Sipilä, Tuomo Kiiskinen, Vincent Llorens, Juulia Partanen, Aki Havulinna, Jiwoo Lee, Kristin Tsuo, Mitja Kurki, Felix Vaura, Jaana Suvisaari, Teemu Niiranen, Veikko Salomaa, Chia-Yen Chen, Sally John, Sanni Lahdenperä, Stephanie Loomis, Susan Eaton, Adam Ziemann, Ali Abbasi, Anne Lehtonen, Apinya Lertratanakul, Bridget Riley-Gillis, Fedik Rahimov, Howard Jacob, Jeffrey Waring, Mengzhen Liu, Nizar Smaoui, Relja Popovic, Athena Matakidou, Benjamin Challis, Dirk Paul, Glenda Lassi, Ioanna Tachmazidou, Adam Platt, George Okafo, Heli Salminen-Mankonen, Marc Jung, Nathan Lawless, Zhihao Ding, Joseph Maranville, Marla Hochfeld, Robert Plenge, Shameek Biswas, David Choy, Edmond Teng, Erich Strauss, Hao Chen, Hubert Chen, Jennifer Schutzman, Julie Hunkapiller, Mark McCarthy, Natalie Bowers, Rion Pendergrass, Tim Lu, Janet Kumar, Margaret G. Ehm, David Pulford, Adriana Huertas-Vazquez, Caroline Fox, Jae-Hoon Sul, Neha Raghavan, Simonne Longerich, Anders Mälarstig, Heli Lehtonen, Jaakko Parkkinen, Kirsi Kalpala, Melissa Miller, Nan Bing, Stefan McDonough, Xinli Hu, Ying Wu, Clément Chatelain, Deepak Raipal, Katherine Klinger, Samuel Lessard, Eric Green, Robert Graham, Sahar Mozaffari, Robert Yang, Alessandro Porello, Amy Hart, Dawn Waterworth, Ekaterina Khramtsova, Karen He, Meijian Guan, Qingqin S. Li, Chris O’Donnell, Ma’en Obeidat, Nicole Renaud, Johanna Schleutker, Antti Hakanen, Nina Pitkänen, Perttu Terho, Petri Virolainen, Auli Toivola, Elina Järvensivu, Essi Kaiharju, Hannele Mattsson, Kati Kristiansson, Lotta Männikkö, Markku Laukkanen, Minna Brunfeldt, Päivi Laiho, Regis Wong, Sami Koskelainen, Sini Lähteenmäki, Sirpa Soini, Terhi Kilpi, Tero Hiekkalinna, Tuuli Sistonen, Jukka Partanen, Mikko Arvas, Anne Pitkäranta, Anu Loukola, Eero Punkka, Malla-Maria Linna, Olli Carpén, Taneli Raivio, Johannes Kettunen, Raisa Serpi, Reetta Hinttala, Tuomo Mantere, Eeva Kangasniemi, Henna Palin, Mika Kähönen, Sanna Siltanen, Tarja Laitinen, Jari Laukkanen, Teijo Kuopio, Johanna Mäkelä, Marco Hautalahti, Outi Tuovila, Raimo Pakkanen, Katriina Aalto-Setälä, Mark Daly, Amanda Elliott, Thomas Damm Als, Masahiro Kanai, Mutaamba Maasha, Wei Zhou, Kristiina Aittomäki, Antti Mäkitie, Natalia Pujol, Triin Laisk, Jarmo Ritari, Kati Hyvärinen, Audrey Chu, Diptee Kulkarni, Fanli Xu, Joanna Betts, John Eicher, Jorge Esparza Gordillo, Laura Addis, Linda McCarthy, Rajashree Mishra, Kirsi Auro, Joni A. Turunen, Aino Salminen, Antti Aarnisalo, Daniel Gordin, David Rice, Erkki Isometsä, Eveliina Salminen, Heikki Joensuu, Ilkka Kalliala, Johanna Mattson, Juha Sinisalo, Jukka Koskela, Kari Eklund, Katariina Hannula-Jouppi, Lauri Aaltonen, Marja-Riitta Taskinen, Martti Färkkilä, Minna Raivio, Oskari Heikinheimo, Paula Kauppi, Pekka Nieminen, Pentti Tienari, Pirkko Pussinen, Sampsa Pikkarainen, Terhi Ollila, Tiinamaija Tuomi, Timo Hiltunen, Tuomo Meretoja, Tuula Salo, Ulla Palotie, Antti Palomäki, Jenni Aittokallio, Juha Rinne, Kaj Metsärinne, Klaus Elenius, Laura Pirilä, Leena Koulu, Markku Voutilainen, Riitta Lahesmaa, Roosa Kallionpää, Sirkku Peltonen, Tytti Willberg, Ulvi Gursoy, Varpu Jokimaa, Kati Donner, Dermot Reilly, Sauli Vuoti, Andrey Loboda, Fabiana Farias, Jason Miller, Anne Remes, Elisa Rahikkala, Johanna Huhtakangas, Kaisa Tasanen, Laura Huilaja, Laure Morin-Papunen, Maarit Niinimäki, Marja Vääräsmäki, Outi Uimari, Peeter Karihtala, Terhi Piltonen, Terttu Harju, Timo Blomster, Vuokko Anttonen, Kai Kaarniranta, Liisa Suominen, Margit Pelkonen, Maria Siponen, Mikko Kiviniemi, Oili Kaipiainen-Seppänen, Päivi Auvinen, Päivi Mäntylä, Debby Ngo, Majd Mouded, Mike Mendelson, Annika Auranen, Airi Jussila, Argyro Bizaki-Vallaskangas, Hannu Uusitalo, Jukka Peltola, Jussi Hernesniemi, Katri Kaukinen, Laura Kotaniemi-Talonen, Pia Isomäki, Teea Salmi, Venla Kurra, Kirsi Sipilä, Fredrik Åberg, Hannu Kankaanranta, Tuula Palotie, Iiris Hovatta, Sanna Toppila-Salmi, Kimmo Palin, Niko Välimäki, Eija Laakkonen, Eija Laakkonen, Eeva Sliz, Heidi Silven, Katri Pylkäs, Minna Karjalainen, Riikka Arffman, Susanna Savukoski, Jaakko Tyrmi, Manuel Rivas, Harri Siirtola, Iida Vähätalo, Javier Garcia-Tabuenca, Marianna Niemi, Mika Helminen, Tiina Luukkaala, Markus Perola, Aarno Palotie, Eero Vuoksimaa, Heiko Runz

**Affiliations:** 1 Institute of Clinical Medicine/Neurology, University of Eastern Finland, Kuopio, Finland; 2 Department of Neurology, Neurocenter, Kuopio University Hospital, Kuopio, Finland; 3 Institute for Molecular Medicine Finland (FIMM), HiLIFE, University of Helsinki, Helsinki, Finland; 4 Department of Neurology, University Medicine Greifswald, Greifswald, Germany; 5 Biobank of Eastern Finland, Kuopio, Finland; 6 Work Ability and Working Careers, Finnish Institute of Occupational Health, Helsinki, Finland; 7 Finnish Institute for Health and Welfare (THL), Helsinki, Finland; 8 Analytic and Translational Genetics Unit, Department of Medicine, Department of Neurology and Department of Psychiatry, Massachusetts General Hospital, Boston, MA USA; 9 The Stanley Center for Psychiatric Research and Program in Medical and Population Genetics, The Broad Institute of MIT and Harvard, Cambridge, MA USA; 10 Translational Sciences, Biogen, Cambridge, MA USA; 11 Abbvie, Chicago, IL USA; 12 Astra Zeneca, Cambridge, UK; 13 Boehringer Ingelheim, Ingelheim am Rhein, Germany; 14 Bristol Myers Squibb, New York, NY USA; 15 Genentech, San Francisco, CA USA; 16 GlaxoSmithKline, Collegeville, PA USA; 17 GlaxoSmithKline, Stevenage, UK; 18 Merck, Kenilworth, NJ USA; 19 Pfizer, New York, NY USA; 20 Translational Sciences, Sanofi R&D, Framingham, MA USA; 21 Maze Therapeutics, San Francisco, CA USA; 22 Janssen Biotech, Beerse, Belgium; 23 Janssen Research & Development, LLC, Spring House, PA USA; 24 Novartis Institutes for BioMedical Research, Cambridge, MA USA; 25 Auria Biobank, Hospital District of Southwest Finland, University of Turku, Turku, Finland; 26 THL Biobank, Finnish Institute for Health and Welfare (THL), Helsinki, Finland; 27 Finnish Red Cross Blood Service, Finnish Hematology Registry and Clinical Biobank, Helsinki, Finland; 28 Helsinki Biobank, Hospital District of Helsinki and Uusimaa, Helsinki University, Helsinki, Finland; 29 Northern Finland Biobank Borealis, Northern Ostrobothnia Hospital District, University of Oulu, Oulu, Finland; 30 Finnish Clinical Biobank Tampere, Pirkanmaa Hospital District, University of Tampere, Tampere, Finland; 31 Central Finland Biobank, Central Finland Health Care District, University of Jyväskylä, Jyväskylä, Finland; 32 FINBB-Finnish Biobank Cooperative, Turku, Finland; 33 Business Finland, Helsinki, Finland; 34 Faculty of Medicine and Health Technology, Tampere University, Tampere, Finland; 35 Aarhus University, Aarhus, Denmark; 36 Department of Medical Genetics, Helsinki University Central Hospital, Helsinki, Finland; 37 Department of Otorhinolaryngology-Head and Neck Surgery, Helsinki University Hospital, University of Helsinki, Helsinki, Finland; 38 Estonian biobank, Tartu, Estonia; 39 Finnish Red Cross Blood Service, Helsinki, Finland; 40 GlaxoSmithKline, Brentford, UK; 41 GlaxoSmithKline, Espoo, Finland; 42 Helsinki University Hospital, University of Helsinki, Helsinki, Finland; 43 Eye Genetics Group, Folkhälsan Research Center, Helsinki, Finland; 44 Hospital District of Helsinki and Uusimaa, Helsinki, Finland; 45 Hospital District of Southwest Finland, Turku, Finland; 46 Janssen Research & Development, LLC, Boston, MA USA; 47 Janssen-Cilag Oy, Espoo, Finland; 48 Merck, Kenilworth, NJ USA; 49 Northern Ostrobothnia Hospital District, Oulu, Finland; 50 Northern Savo Hospital District, Kuopio, Finland; 51 Novartis, Basel, Switzerland; 52 Novartis, Boston, MA USA; 53 Pirkanmaa Hospital District, Tampere, Finland; 54 Research Unit of Oral Health Sciences Faculty of Medicine, University of Oulu, Oulu, Finland; 55 Medical Research Center, Oulu University Hospital, University of Oulu, Oulu, Finland; 56 Transplantation and Liver Surgery Clinic, Helsinki University Hospital, Helsinki University, Helsinki, Finland; 57 University of Gothenburg, Gothenburg, Sweden; 58 Seinäjoki Central Hospital, Seinäjoki, Finland; 59 University of Stanford, Stanford, California USA; 60 Hospital District of Helsinki and Uusimaa, University of Helsinki, Helsinki, Finland; 61 University of Helsinki, Helsinki, Finland; 62 University of Jyväskylä, Jyväskylä, Finland; 63 University of Oulu, Oulu, Finland

**Keywords:** Biomarkers, Neurology

## Abstract

Successful development of novel therapies requires that clinical trials are conducted in patient cohorts with the highest benefit-to-risk ratio. Population-based biobanks with comprehensive health and genetic data from large numbers of individuals hold promise to facilitate identification of trial participants, particularly when interventions need to start while symptoms are still mild, such as for Alzheimer’s disease (AD). This study describes a process for clinical recall studies from FinnGen. We demonstrate the feasibility to systematically ascertain customized clinical data from FinnGen participants with ICD10 diagnosis of AD or mild cognitive disorder (MCD) in a single-center cross-sectional study testing blood-based biomarkers and cognitive functioning in-person, computer-based and remote. As a result, 19% (27/140) of a pre-specified FinnGen subcohort were successfully recalled and completed the study. Hospital records largely validated registry entries. For 8/12 MCD patients, other reasons than AD were identified as underlying diagnosis. Cognitive measures correlated across platforms, with highest consistencies for dementia screening (r = 0.818) and semantic fluency (r = 0.764), respectively, for in-person versus telephone-administered tests. Glial fibrillary acidic protein (GFAP) (p < 0.002) and phosphorylated-tau 181 (pTau-181) (p < 0.020) most reliably differentiated AD from MCD participants. We conclude that informative, customized clinical recall studies from FinnGen are feasible.

## Introduction

Alzheimer’s disease (AD) is the most common neurodegenerative condition and a major socio-economic challenge. It has been estimated that during the next four decades the prevalence of AD will quadruple from 27 to 106 million by which time 1 in 85 individuals worldwide will be living with the disease. Even a modest delay of disease onset could reduce the number of cases substantially^1^. Interventions for AD are expected to be most efficacious when initiated in prospective patients at the earliest stages of disease^2^. However, due to the lack of curative therapies or easily measurable biomarkers, it is current clinical practice that a majority of the patients receives their diagnosis only at progressed stages when irreversible alterations to brain and cognitive performance have already occurred. There is thus a substantial interest across industry and academia to identify AD patients early, and to align on measures that reliably diagnose the disease at the earliest possible stages to refine its onset and predict its course and response to experimental treatments.

Population-based, large-scale biobank studies that link detailed longitudinal health information with genetic and biomarker data are expediting the development of tools to identify individuals with a high probability to develop AD and other late onset diseases^3–5^. They further hold the promise to accelerate recruitment of newly diagnosed patients or individuals at risk into observational and interventional clinical trials, or to facilitate access to efficient treatments^6,7^. However, thus far few studies have overcome the scientific, logistical, and ethical challenges to recall individuals consented for broad population-level biobank research into customized clinical follow-up studies based on their individual-level health, genetic, or biomarker data.

The FinnGen (FG) study (https://www.finngen.fi) is a precompetitive partnership between thirteen industry partners and the Finnish biobanks to genetically profile and link genetics with detailed biomedical and social information in 10% of the Finnish population. Since its inception in 2017, by 2023 FG has recruited over 500,000 participants with registry data on more than 2000 harmonized disease endpoints and contributed to numerous scientific discoveries^8,9^. FG participants are broadly consented for secondary use of research data and in principle re-contactable for follow-up studies through participating biobanks.

Here, we established a process for recalling FG participants fulfilling distinct, pre-specified inclusion criteria into a clinical follow-up study to obtain customized novel clinical and biomarker data. In a single-center, cross-sectional pilot study, we targeted a population that based on registry entries had been diagnosed (ICD10) with either AD or mild cognitive disorder (MCD). We assessed the accuracy of FG registry versus hospital records, applied three different approaches to evaluate cognitive functioning via in-person neuropsychological assessment, computerized and telephone-administered testing, and obtained blood-based AD biomarkers. Our results validate the accuracy of clinical information captured in Finnish registries, support remote cognitive testing as an effective screening approach, and demonstrate that recall studies from FG are feasible.

## Materials and methods

### Study participant selection

**Figure 1:**
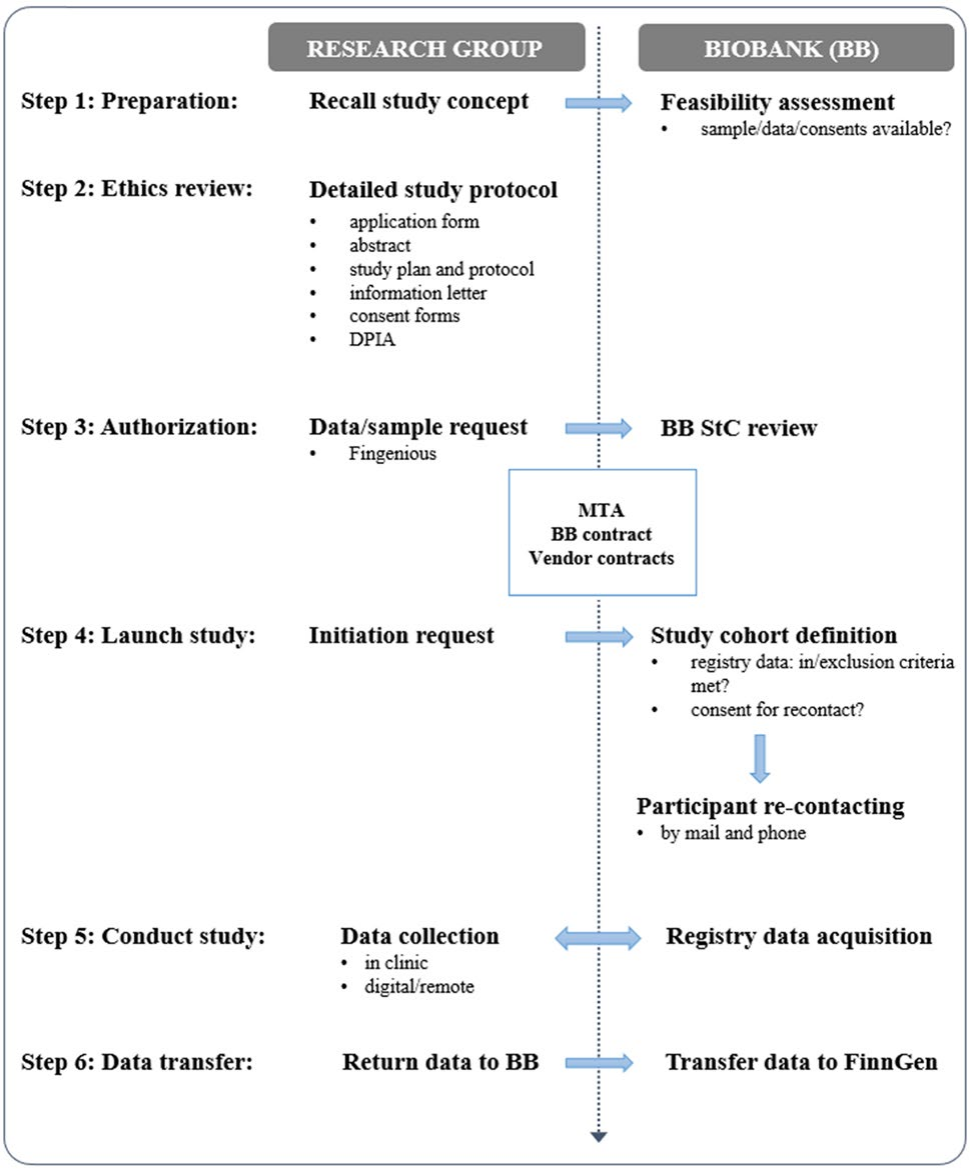
Operationalization of this clinical recall study from FinnGen. *DPIA* data protection impact assessment, *BB* biobank, *MTA* material transfer agreement, *StC* steering committee.

**Figure 2:**
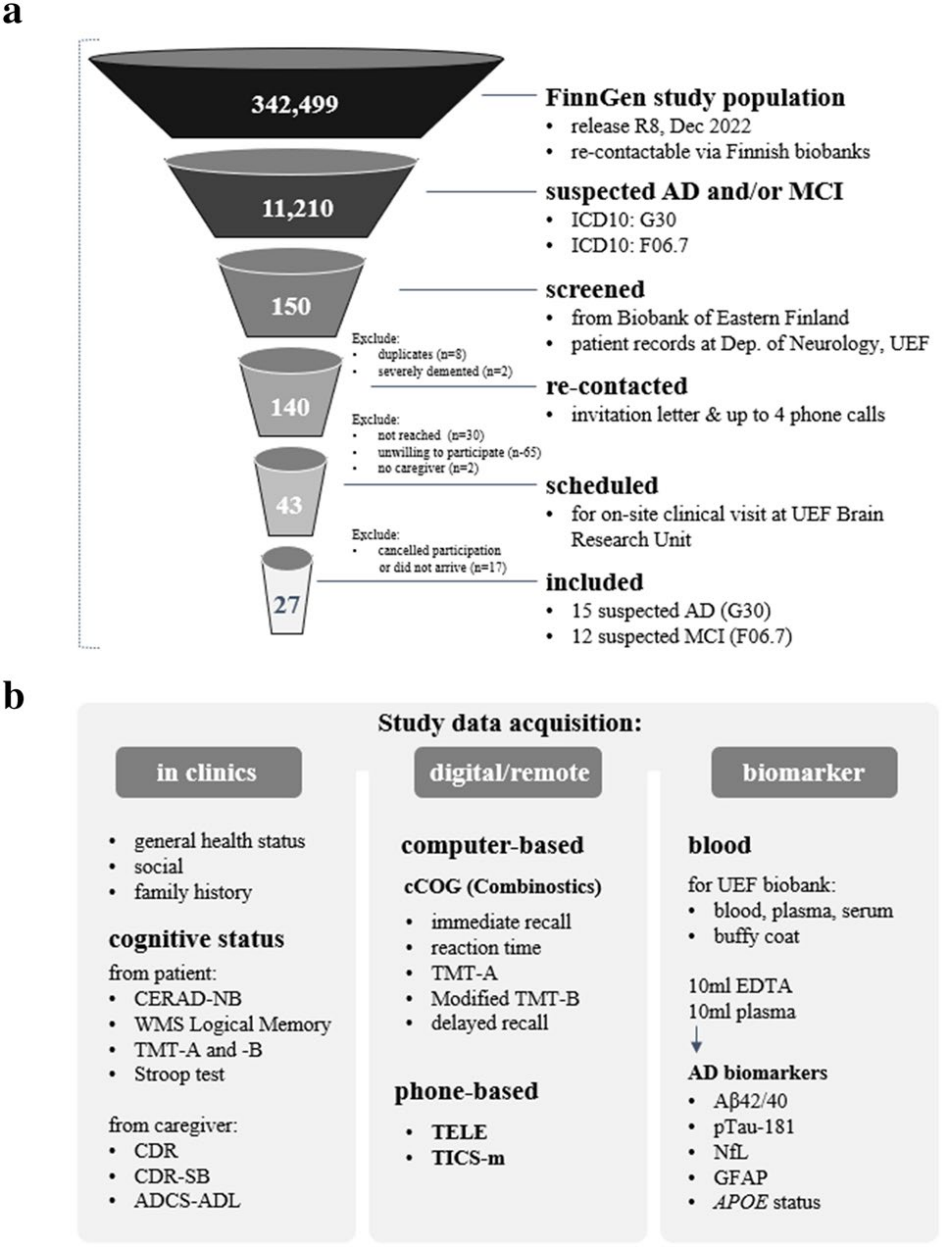
(**a**) Screening and enrollment process for study. *AD* Alzheimer’s disease, *MCD* mild-cognitive disorder, *UEF* University of Eastern Finland. (**b**) New data acquired during study. *CERAD-NB* Consortium to Establish a Registry for Alzheimer’s Disease Neuropsychological Battery, *WMS* Wechsler Logical Memory Scale, *TMT-A/B* trail making test parts A and B, *CDR* clinical dementia rating scale, *CDR-SB* CDR sum of boxes, *ADCS-ADL* Alzheimer’s Disease Cooperative Study-Activities of Daily Living scale, *TELE* telephone assessment for dementia, *TICS-m* modified telephone interview for cognitive status, *Aβ-42/40* amyloid beta (Aβ)1–42/1–40, *p-Tau181* phosphorylated-tau181, *NfL* neurofilament light chain, *GFAP* glial fibrillary acidic protein;

For this pilot study we targeted a FG subcohort with registry-defined diagnosis of either a wide definition of AD (ICD10: G30), or a registry entry of the ICD10 code F06.7 “Mild neurocognitive disorder due to known physiological condition”, the closest diagnostic code to mild cognitive impairment, a heterogenous condition frequently used to describe individuals with a higher probability to develop AD. Moreover, study participants were required to have provided consent for recontact, as well as for sample and data usage to the Biobank of Eastern Finland (BEF). The process for accessing biobank data and steps required to operationalize a biobank recall study is guided by the Finnish biobank regulations (https://ita-suomenbiopankki.fi/en/researchers/) and visualized in Fig. 1. In brief, after a positive feasibility assessment by BEF, approval of the study protocol and accompanying documents by the ethics committee of Helsinki University Hospital, and ensuring a permit to access patient files from Kuopio University Hospital (KUH), a request for biobank data access was launched via the Fingenious portal (https://site.fingenious.fi/). This triggered a formal evaluation of the study plans by the BEF scientific steering committee and initiation of contracts between participating parties. An updated feasibility assessment by BEF resulted in 150 participants meeting study inclusion criteria. Of these, eight were removed as they were identified as duplicates (same person counted twice in the registry). Two additional individuals were excluded since they lived in institutionalized care with a likely late-stage AD and not being able to travel to the study site. The study participant selection with exclusions is presented in Fig. 2a.

A total of 140 FG participants were contacted by BEF personnel. Recontact was conducted by using a letter that included study information and forms for informed written consent. To ensure that the study information had reached the potential study participants, and to further enhance the participation rate, all potential participants received a phone call from the BEF study nurse within 1–2 weeks after sending the invitation material. During this phone call the potential participants received standard structured information concerning the study, were asked whether they were willing to participate, and a date for on-site assessment at the University of Eastern Finland (UEF) Brain Research Unit (BRU) was scheduled. Participants willing to participate returned their informed written consents either by mail to BRU, or by bringing the signed forms to their appointment. Participants who completed the in-person and computerized cognitive testing batteries at the BRU received a follow-up phone-call after 1–2 weeks with the aim to perform a remote telephone-administered cognitive assessment. Data obtained during this study are summarized in Fig. 2b.

### Schedule of assessment

All study participants were invited to UEF-BRU for: a structured interview of the study participant and caregiver or family member to obtaining basic patient information and to fill out questionnaires regarding abilities of daily life and functioning; drawing of blood samples for AD biomarker profiling and optional future biomarker discovery; in-person neuropsychological assessment performed by a trained psychologist; and on-site computer-administered cognitive function testing. Moreover, cognitive function was subsequently evaluated by phone-interview 1–2 weeks later. All cognitive assessments were done in Finnish language. Half of the study participants were randomized to obtain computerized-testing before in-person testing to reduce the risk of systematic bias from practice effect.

### Data linkage to FinnGen and hospital records

In addition to obtaining new clinical and biomarker data, we obtained approval to link these data to previously ascertained health care register data such as hospital diagnoses, medication records, social registry entries, and genetic data. Data linkage was performed in a controlled FG Sandbox environment approved to host individual-level data and accessible only by approved researchers. In addition to data directly ascertained through the study protocol or available through FG, we also analyzed earlier routine care data available for study participants through the patient records from KUH. This data included clinical information from earlier memory clinic and follow-up visits (e.g., historic cognition test results), previous laboratory values (e.g., results from cerebrospinal fluid examinations for AD biomarkers), and neuroimaging data (e.g., magnetic resonance imaging ascertained through routine care). Among others, data linkage offered the possibility to compare the accuracy of FG registry data against hospital records and validate that inclusion criteria were indeed met based on earlier clinical information.

### In-person neuropsychological testing

In-person neuropsychological testing included the Consortium to Establish a Registry for Alzheimer’s Disease Neuropsychological Battery (CERAD-NB)^10^. Additional tests included Logical Memory subtest from the Wechsler Adult Intelligence Scale 3rd edition^11^, Trail Making Test A (TMT-A) and B (TMT-B)^12^, and a short 40-item version of the Stroop test^13^. The tests were administered by a trained psychologist at the UEF-BRU. For the current study we investigated the following four specific cognitive domains (with specific measures in parentheses): episodic memory (immediate free recall of words in trials 1–3 and delayed free recall of the 10-word list from the CERAD-NB); executive function (set-shifting: TMT-B controlled for TMT-A); processing speed (TMT-A); and semantic fluency (1-min animal naming).

In the 10-word list learning participants were shown 10 words. Word list was presented visually and verbally three times and after each trial participants were asked to name all words that they remembered. In the delayed free recall (after 5 min from the immediate recall), participants were again asked to name all words that they remembered. In TMT-A, participants were asked to connect numbered circles from smallest to biggest (1, 2, 3, etc.) and in TMT-B participants were asked to connect circles with numbers and alphabets by alternating between these two (1, A, 2, B, etc.). Time to complete TMT-A and TMT-B was recorded and both conditions had practice trials. In animal naming, participants were asked to name as many animals as they can in one minute.

Word list learning task and animal naming from the CERAD-NB and TMT-A/B measures were selected for this study because similar or corresponding measures were also available in computerized testing and in the telephone interview (described below). In addition, we used Mini-Mental State Examination from the CERAD-NB in comparison with telephone administered cognitive screening measure described below.

These measures were selected because similar or corresponding measures were also available in computerized testing and in the telephone interview (described below).

### Computerized cognitive testing

We used the cCOG^14^ platform developed by Combinostics Oy (https://www.cneuro.com), a self-administered computer-based testing tool for assessing cognitive performance at an on-site computer via a keyboard and a mouse. A total of seven tasks measured performance in four specific cognitive domains: episodic memory, executive function, processing speed, and reaction time. An earlier study proposed that cCOG has comparable accuracy to in-person neuropsychological batteries for detecting dementia, and at least moderately correlates with specific in-person neuropsychological tests^14^. In this current study, we used episodic memory measures of immediate (total words recalled in trials 1–3) and delayed recall of 12-word list measures. We also used the Modified TMT, with two parts corresponding to the traditional TMT-A and -B, to measure processing speed and executive function (set-shifting). Detailed test descriptions and protocols can be found in Rhodius-Meester et al. (2020)^14^. Completing cCOG took participants about 25 min.

### Telephone interview to assess cognitive performance

We used two validated, telephone-administered cognitive screening instruments: telephone assessment for dementia (TELE)^15^ and the modified Telephone Interview for Cognitive Status (TICS-m)^16^. Telephone interviews took on average 17 (range 12–33) minutes with an average of 13 (range 9–29) minutes to complete TELE and TICS-m and were conducted by a trained research nurse. For analyses in the current study, we used the following measures: For TELE, we used a continuous score (0–20) and validated cut-offs for cognitive impairment (< 16) and healthy cognition (> 17.5)^17^. From TICS-m, we used episodic memory measures of verbal immediate (total words recalled in trials 1–3) and delayed free recall of 10-word list. In the original TICS-m, there is only a single trial of the 10-word list in the learning condition. Here we modified this task to include three learning trials in order to make this measure more similar to in-person and computerized list learning tasks. In addition, semantic fluency was measured (animals in 1-min).

### Assessment of functional status and independence in activities of daily living

The severity of cognitive symptoms and functioning disability was estimated by the clinical dementia rating scale (CDR) and CDR sum of boxes (CDR-SB)^18^ as well as by the 18-item Alzheimer’s Disease Cooperative Study-Activities of Daily Living scale (ADCS-ADL)^19^. This on-site assessment was done by a trained study nurse and included an interview of the caregiver or family member of the study participant to increase the reliability of data acquisition.

### AD blood-based biomarkers and *apolipoprotein E* (*APOE*) genotype

The analyses of plasma biomarkers phosphorylated-tau181 (pTau-181), amyloid beta (Aβ)1–40, Aβ1-42, glial fibrillary acidic protein (GFAP) and neurofilament light chain (NfL) from all 27 patients were performed using Simoa HD-X analyzer (Quanterix, Billerica, Massachusetts, USA), which employs single molecule array digital immunoassay technology. Analyses were performed at the biomarker laboratory of UEF-BRU. Plasma pTau-181 levels were quantified using Simoa pTau-181 Advantage V2 Kit (Ref# 103714, Quanterix) and Aβ1-40, Aβ1-42, GFAP and NfL levels using Simoa Neurology 4-Plex E Advantage Kit (Ref# 103670, Quanterix) according to the manufacturer's instructions. Prior to analyses, EDTA plasma samples were thawed, mixed, centrifuged (10,000×*g*, 5 min, + 20 °C) and measured in duplicates. All replicates had coefficient of variation below 15%. *APOE* status was defined by two single-nucleotide polymorphisms, rs429358 and rs7412, in chromosome 19. We classified individuals as Σ4-carriers vs. non-carriers.

### Statistical analyses

We compared AD and MCD groups with regards to cognitive function and performed comparisons between in-person, computerized and telephone-administered tests. Cognitive screening instruments were the Mini Mental State Examination (MMSE) as a part of CERAD-NB battery administered in-person and TELE included in the telephone interview. We also classified individuals in cognitively impaired versus cognitively intact based on the CERAD-NB word list learning immediate and delayed recall measures. With regards to specific abilities, we compared performance in the following cognitive domains (measured on at least two platforms): episodic memory, executive function (set-shifting), processing speed, and semantic fluency. Episodic memory was measured in all three formats (CERAD word list in in-person, TICS 10-word list via telephone and 12-word list in cCOG), processing speed and executive function in two formats (TMT-A & TMT-B in in-person and modified TMT-A & TMT-B in cCOG), and semantic fluency in two formats (1-min animal fluency both in in-person and telephone testing). Statistical analyses were performed with R version 4.1.1 (available online at https://www.R-project.org/). Spearman’s rank partial correlation analyses were used to test the associations between in-person, computerized and telephone-administered tests. We report Spearman correlations due to small sample size and non-normal distribution of some of the measures. In addition, cognitive test results, age, and the time from original diagnosis to study visit were compared between AD and MCD groups using independent t-tests. The significance level was set at α = 0.05.

### Ethics approvals

All participants in this recall study were also participants in FG and had provided informed consent for biobank research including an option for recalling into a new scientific study. This procedure is based on the Finnish Biobank Act. All recall study participants and their respective caregiver or family member received detailed information regarding this study by a formal information letter, as well as structured information provided during a phone call. All study participants and their participating caregivers were asked for and provided written informed consent. All study data was initially stored locally at UEF (on-site assessment) and the Finnish Institute for Molecular Medicine (FIMM) (remote cognition assessment by phone). Study results and data were then returned first to BEF and from there further to the FG Sandbox controlled environment to link newly ascertained clinical data with genetic and register data. The recall study was reviewed and approved by the ethics committee of HUS under IRB approval number 990/2017. Additionally, research permissions were granted from BEF (diary ID 1248/2021) and KUH (1535/2021, study ID 5772671). All experiments were performed in accordance with relevant guidelines and regulations of these instances and the Declaration of Helsinki.

An alternative process for dedicated biobank consent has been applied for a subset of FG participants. Some of the separate legacy research cohorts collected prior the Finnish Biobank Act came into effect (in September 2013) and start of FG (August 2017) were collected based on previous study-specific consents and later transferred to the Finnish biobanks and eventually to FG after approval by Fimea (Finnish Medicines Agency) and the National Supervisory Authority for Welfare and Health. Recruitment protocols followed the biobank protocols approved by Fimea. The Coordinating Ethics Committee of the Hospital District of Helsinki and Uusimaa (HUS) statement number for the FG study is Nr HUS/990/2017. The permit numbers of the decisions made by Finnish Institute for Health and Welfare and the Biobank Access Decisions for FG samples and data utilized in FG Data Freeze 8 are presented in Acknowledgements.

## Results

### Operationalization of a FinnGen clinical recall study

FG data has been accumulating steadily since 2017, reaching over 500,000 consented individuals by the year 2023. At the time of study (October 2021-February 2022), FG had ascertained genetic and registry data on 342,499 participants (190,879 females, 151,620 males; Data Freeze 8, publicly released in December 2022 via http://finngen.fi/en/accesss_results). Of these, health records from 11,210 participants were characterized by at least one entry of the FG diagnostic code “G6_ALZHEIMER” (curated based on ICD10 code G30), reflecting a putative diagnosis of AD. 2170 participants had at least one entry for ICD10 code F06.7, reflecting a diagnosis of MCD (http://risteys.finngen.fi/). For this proof-of-concept recall study, we decided to focus on participants recruited into FG from just one of the nine contributing biobanks, Biobank of Eastern Finland (BEF; http://ita-suoimenbiopankki/en/). To enable comparison of FG registry data with individual-level health records and limit geographic outreach, we further requested that study participants were still alive, lived within a proximity of 100 km from the study site and had earlier been a patient at the Department of Neurology, KUH.

A flow-chart visualizing study participant selection and data ascertained during study are provided in Fig. 2, and the process how this FG recall study was operationalized is described in Fig. 1 and Methods. In brief, over a period of 15 business days, 277 phone calls were made (maximum of 3–4 calls/person) of which 151 were not answered. A total of 65 individuals were reached and declined to participate, 30 individuals were not reached, and for two individuals no caregiver could be identified to accompany the in-principle-interested participant to a near-term on-site visit. This resulted in a total of 43 FG participants (40% of the 108 reached and eligible) for whom a study visit at the UEF-BRU was scheduled. A total of 27 on-site visits were performed in November and December 2021. The initial plan to continue recruitment of this particularly vulnerable population until the end of February 2022 was discontinued after the majority of scheduled visits were canceled at the peak of a SARS-Cov2 outbreak in Finland in early 2022. With this, 63% (27/43) of the scheduled participants came to study visit and the participation rate of this first FG clinical recall study was 19% (27/140) when considering the entire a-priori eligible and re-contactable cohort, but it likely would have been substantially higher if all scheduled study visits could have been completed in 2021.

### Registry diagnoses and hospital records are largely consistent

Of the 27 FG participants included in the study, 13 were females (Table 1). The mean age at study visit was 73 (58–87) years. Based on FG registry data, 15 study participants had a diagnosis of AD, while 12 participants had been diagnosed with MCD. Mean age at the first diagnostic entry of either AD or MCD was 69 (50–81) and 69 (58–80), respectively, with the mean time since diagnosis being 4 (1–13) years for AD and 3 (1–6) years for the MCD patients under study. More detailed information about demographic factors (education, smoking, alcohol usage and comorbidities) are available in Supplementary table (Table S1). Based on these measures, our subcohort was largely representative of the broader FG AD and MCD populations (http://risteys.finngen.fi/).

**Table 1:** Characteristics of the study cohort.

	AD (n = 15)	MCD (n = 12)	p-value
Age (years)	73.0 (8.8)	72.5 (8.3)	0.895
Sex, females/males (n)	8/7	5/7	0.830
APOE4 carrier (n)	10	3	0.171
MMSE (score)	24.0 (7.6)	24.1 (6.7)	0.977
In-person tests			
CERAD immediate recall (score)	14.6 (6.6)	15.2 (6.0)	0.812
CERAD delayed recall (score)	4.0 (2.8)	5.0 (2.4)	0.343
TMT-A (s)	84.3 (54.2)	83.6 (35.0)	0.973
TMT-B (s)	178.1 (83.9)	215.2 (83.8)	0.337
Semantic fluency (score)	18.1 (7.3)	18.0 (8.2)	0.963
Computer-based tests			
cCOG immediate recall (score)	13.6 (6.2)	12.6 (5.1)	0.666
cCOG delayed recall (score)	3.5 (3.2)	4.1 (2.8)	0.632
cCOG TMT-A modified (s)	98.8 (74.1)	81.1 (33.6)	0.567
cCOG TMT-B modified (s)	231.6 (87.4)	261.0 (91.6)	0.525
Phone-based tests			
TELE total (score)	15.3 (4.9)	17.2 (4.9)	0.346
TICS-m immediate recall (score)	14.5 (9.6)	14.2 (6.6)	0.927
TICS-m delayed recall (score)	3.8 (4.0)	2.6 (2.4)	0.365
Semantic fluency (score)	15.1 (6.8)	17.2 (6.7)	0.446

Data are given as mean and standard deviation for Alzheimer’s disease (AD) and mild cognitive disorder (MCD) groups separately. *APOE*4 carrier status was available for 23 (14AD/9MCD) and MMSE, CERAD immediate and delayed recall, cCOG immediate and delayed recall, and in-person verbal fluency data was available for 26 (14AD/12MCD) participants. In-person TMT-A was available for 25 (14AD/11MCD), in-person TMT-B for 20 (11AD/9MCD), cCOG TMT-A for 17 (10AD/7MCD), and cCOG TMT-B for 17 (11AD/6MCD) participants.

*APOE* apolipoprotein E, *cCOG* computerized cognitive testing, *CERAD* Consortium to Establish a Registry for Alzheimer’s Disease, *MMSE* Mini Mental State Examination, *TELE* telephone assessment for dementia, *TICS-m* modified telephone interview for cognitive status with three learning trials of the 10-word list, *TMT-A/B* trail making test parts A and B.

For all participants, the dates of diagnosis between FG registry entry and hospital records were within a six-month range. For one participant, F06.7 was only documented in registry, but not in hospital records. According to hospital memory clinic records, only a single patient had progressed to AD from an initial diagnosis of MCD. For the majority of the MCD group, clinical interpretation of the hospital records could pinpoint at least one alternative etiology explaining the mild cognitive impairment. This included one case with vascular cognitive impairment, one with cortico-basal degeneration and one with idiopathic normal pressure hydrocephalus. Other putative reasons for MCD included Parkinson's disease, depression, epilepsy, menopause, sleeping problems, or tiredness due to poor overall health. Conversely, for 13 of the 15 patients in the AD group, the clinical diagnosis had been based on current Finnish diagnostic guidelines for memory disorders^20^, which included administration of a formal neuropsychological testing battery (CERAD-NB or comprehensive neuropsychological assessment), brain imaging with magnetic resonance imaging or computed tomography, thorough neurological and physical examination as well as in several cases cerebrospinal fluid biomarkers and brain positron emission tomography. Two participants in the AD group did not have diagnostic information in hospital records. For these cases, the validity of a prior diagnosis of AD could not be confirmed. Three had documentation for mixed AD: in one case AD presented together with vascular dementia features, in another AD was documented together with Lewy-Body dementia, and one case was documented to have a posterior variant of AD.

### In-person, computerized and telephone-administered assessment of cognition

Data acquisition via all three platforms was in general feasible for participants, with no substantial differences between the AD and MCD groups, although several individuals from both groups were unable to complete specific subtests. Most problems occurred with the computerized versions of TMT-A and TMT-B which were completed by only 17 participants.

In-person MMSE scores could be obtained from 26 participants. Of these, 17 (8AD/9MCD) showed normal cognitive function or only mild impairment (MMSE ≥ 25), while 9 (6AD/3MCD) were classified as cognitively impaired (MMSE < 25). Although the mean MMSE scores did not differentiate between AD (24.0 ± 7.6) and MCD (24.1 ± 6.7) groups (p = 0.977, Table 1), there were more cognitively impaired individuals in the AD group (43%, 6/14 versus 25%, 3/12 for MCD).

According to TELE, 16 participants (8AD/8MCD) were classified as cognitively healthy (cut-off score > 17.5), while 7 (5AD/2MCD) participants were classified as cognitively impaired (cut-off score < 16), and 4 (2AD/2MCD) participants ranked between the cut-off scores (< 17.6 and ≥ 16). On average those with AD (15.3 ± 4.9) had non-significantly (p = 0.345) lower scores than those with MCD (17.2 ± 4.9). The prevalence of cognitively impaired was almost double in the AD group (33%, 5/15) than in the MCD group (17%, 2/12).

Based on CERAD-NB word list immediate recall, 71% (10/14) of those with AD and 50% (6/12) of those with MCD were classified as having episodic memory impairment (cut-off < 17), with mean immediate recall scores of 14.6 ± 6.6 and 15.2 ± 6.0 in AD and MCD subgroups, respectively (p = 0.812). Based on the CERAD-NB word list delayed recall, 57% (8/14) of those with AD and 33% (4/12) of those with MCD were classified as having episodic memory impairment (cut-off < 5), with mean scores of 4.0 ± 2.8 and 5.0 ± 2.4 in AD and MCI subgroups, respectively (p = 0.343).

**Figure 3:**
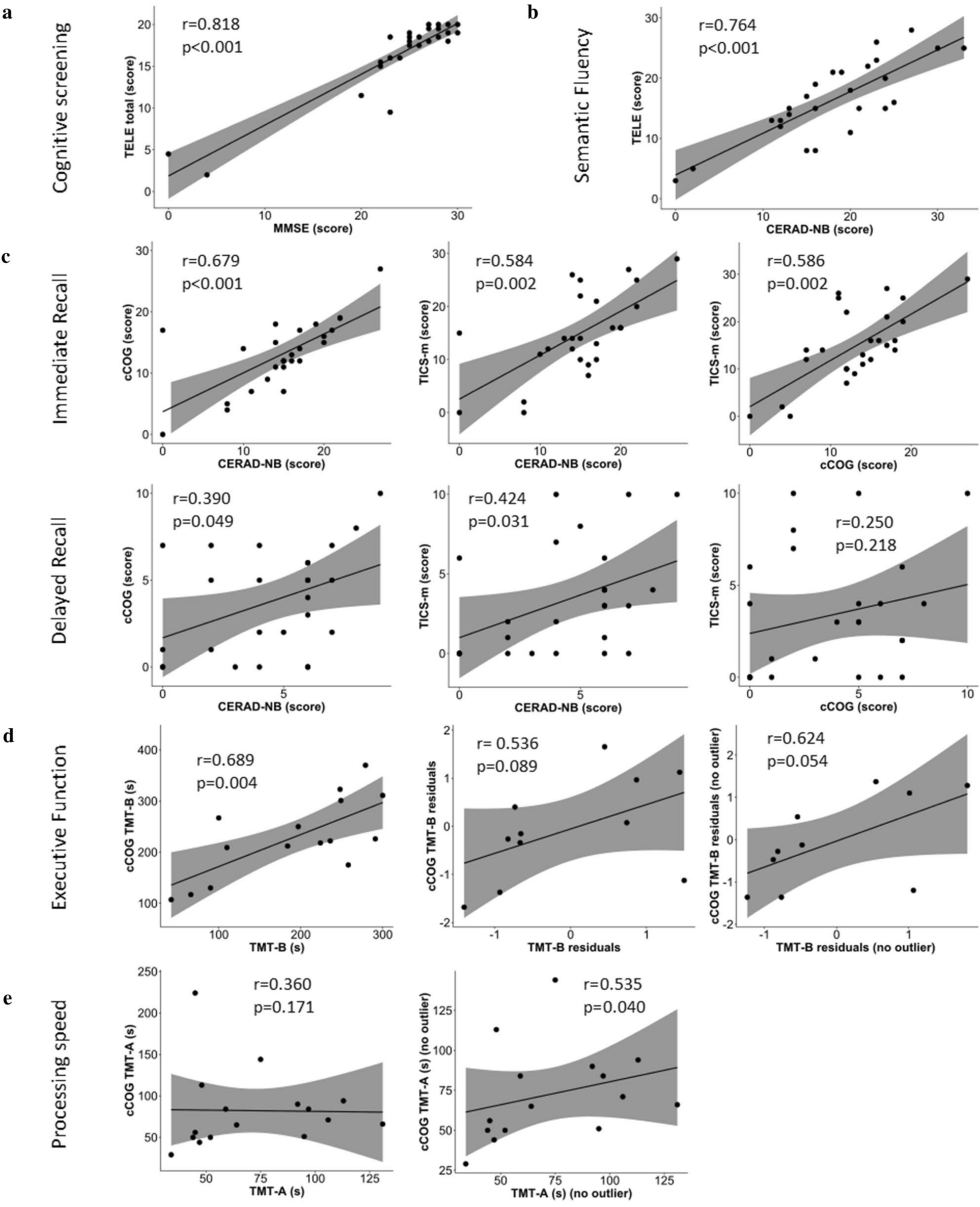
Comparison of cognitive testing results across the three platforms utilized in the study. Correlations of (**a**) cognitive screening, (**b**) semantic fluency, (**c**) immediate and delayed recall, and (**d**) executive function are presented between in-person, computerized, and telephone-based assessments. Scatterplots with 95% confidence intervals (shaded areas) display correlation coefficient r and p-value from Spearman’s rank partial correlation analyses. *cCOG* computerized cognitive testing, *CERAD-NB* Consortium to Establish a Registry for Alzheimer’s Disease Neuropsychological Battery, *MMSE* Mini Mental State Examination, *TELE* telephone assessment for dementia, *TICS-m* modified telephone interview for cognitive status with three learning trials of the 10-word list, *TMT-A/B* trail making test parts A and B.

Continuous MMSE and TELE scores showed a high correlation of r = 0.818 (p < 0.001) (Fig. 3a, Table 1). A similar high correlation was observed for semantic fluency as measured by in-person testing as well as a part of the telephone interview (r = 0.764; p < 0.001; Fig. 3b). Considering measures of episodic memory, immediate word recall measures were positively correlated between all three platforms (all r’s ≥ 0.584; p’s ≤ 0.002). Correlations between delayed word recall measures were smaller, with CERAD-NB showing modest correlations with cCOG (r = 0.390, p = 0.049) and TICS-m (r = 0.424, p = 0.031) whereas the correlation between cCOG and TICS-m measures was small (r = 0.250, p = 0.218) (Fig. 3c). Strong correlations were observed between in-person and computerized measures of executive function (TMT-B; r = 0.689, p = 0.004, n = 15; r = 0.536, p = 0.089, n = 11 after adjusting for TMT-A and r = 0.624, p = 0.054, n = 10 after additional exclusion of TMT-A outliers) (Fig. 3d**)**, whereas in-person versus computerized measures of processing speed correlation was moderate to strong (TMT-A; r = 0.360, p = 0.171, n = 16; r = 0.535, p = 0.40, n = 15 after exclusion of outliers) (Fig. 3e).

### Blood-based biomarkers and *APOE* genotype

Across all blood-based biomarker parameters, values varied substantially (Table 2). Notably, despite the small cohort size we observed statistically significant differences between the AD and MCD groups for GFAP [p = 0.002; AD: 216.3 (57.5–433.7) pg/ml versus MCD: 102.8 (30.5–161.3) pg/ml] and pTau-181 [p = 0.020; AD: 3.8 (1.2–11.7) pg/ml versus MCD: 1.9 (0.6–3.6) pg/ml], whereas no significant differences were seen for Aβ42/40-ratio (p = 0.266) or NfL (p = 0.140). TELE total score was significantly correlated with GFAP (p = 0.011), NfL (p = 0.001) and pTau-181 (p = 0.006), but not with Aβ42/40 (p = 0.345), whereas non-significant correlations of MMSE score with biomarkers ranged from -0.247 to 0.020. With the exception of correlations between pTau-181 levels and telephone-administered immediate recall of the word list (r = -0.394, p = 0.042, n = 27) and in-person administered TMT-A (r = 0.435, p = 0.034, n = 24) no other correlations between biomarkers and cognitive performance measures reached statistical significance threshold.

**Table 2:** Blood-based biomarkers in study participants with Alzheimer’s disease (AD) and mild cognitive disorder (MCD) diagnosis and correlations with cognitive measures.

	Aβ42/40	GFAP (pg/ml)	NfL (pg/ml)	pTau-181 (pg/ml)
AD (n = 15), mean (SD) & range	0.055 (009) 0.035–0.071	216.3* (107.7) 57.5–433.7	24.6 (15.3) 11.2–73.8	3.8* (2.6) 1.2–11.7
MCD (n = 12), mean (SD) & range	0.059 (0.011) 0.031–0.072	**102.8 (39.8)** 30.5–161.3	17.3 (7.4) 9.2–28.1	**1.9 (0.9)** 0.6–3.6
r (p-value) with cognitive screening instruments				
MMSE (n = 26)	0.020 (0.923)	−0.146 (0.476)	−0.220 (0.280)	−0.247 (0.223)
TELE (n = 27)	0.189 (0.345)	**−0.480 (0.011)**	**−0.587 (0.001)**	**−0.511 (0.006)**
r (p-value) with episodic memory tests (immediate and delayed recall measures)				
CERAD immediate (n = 26)	0.156 (0.446)	−0.149 (0.468)	−0.327 (0.103)	−0.202 (0.322)
CERAD delayed (n = 26)	0.323 (0.108)	−0.211 (0.300)	−0.190 (0.353)	−0.254 (0.210)
cCOG immediate (n = 26)	0.064 (0.757)	−0.178 (0.383)	−0.259 (0.202)	−0.222 (0.276)
cCOG delayed (n = 26)	0.195 (0.341)	−0.338 (0.091)	−0.177 (0.387)	−0.300 (0.136)
TICS-m immediate (n = 27)	0.051 (0.802)	−0.172 (0.392)	−0.280 (0.158)	−**0.394 (0.042)**
TICS-m delayed (n = 27)	−0.002 (0.994)	−0.091 (0.650)	−0.108 (0.592)	−0.300 (0.129)
r (p-value) with processing speed				
TMT-A (n = 24)	0.044 (0.837)	0.281 (0.184)	0.344 (0.099)	**0.435 (0.034)**
cCOG TMT-A modified (n = 15)	0.233 (0.404)	0.020 (0.945)	0.118 (0.675)	0.245 (0.379)
r (p-value) with set-shifting (adjusted for processing speed)				
TMT-B (n = 20)	−0.198 (0.402)	0.125 (0.600)	0.095 (0.691)	0.095 (0.691)
cCOG TMT-B modified (n = 11)	−0.118 (0.729)	−0.264 (0.433)	−0.327 (0.326	−0.018 (0.958)
r (p-value) with semantic fluency (SF)				
SF in-person (n = 26)	0.031 (0.880)	−0.072 (0.727)	−0.302 (0.134)	−0.088 (0.668)
SF telephone (n = 27)	0.188 (0.348)	−0.171 (0.395)	−0.324 (0.100)	−0.281 (0.156)

Significant values are in [bold].

*AD* Alzheimer’s disease, *Aβ42/40* amyloid beta 42/40 ratio, *cCOG* computerized cognitive testing, *CERAD* Consortium to Establish a Registry for Alzheimer's Disease, *GFAP* glial fibrillary acidic protein, *MCD* mild cognitive impairment, *MMSE* Mini Mental State Examination, *NfL* neurofilament light chain, *pTau-181* phosphorylated-tau181, *TELE* telephone assessment for dementia, *TICS-m*, modified telephone interview for cognitive status with three learning trials of the 10-word list, *SD* standard deviation, *TMT-A/B* trail making test parts A and B, *VF* verbal fluency.

*Significantly higher value in AD group in comparison to MCD group. Participants with > 200 in TMT-A (modified) were excluded from the processing speed and set-shifting correlations.

*APOE* status was available for 23 participants. Of these 13 were *APOE* Σ4-carriers (12 heterozygotes, 1 homozygote). There were 71% (10/14) and 33% (3/9) *APOE* Σ4-carriers (p = 0.072) in the AD and MCD groups, respectively (p = 0.072 for the difference).

## Discussion

In this single-center, cross-sectional pilot study we ascertained clinical-grade cognitive function and biomarker data from a pre-specified FG subcohort in order to build a framework to operationalize future clinical recall studies from FG. We demonstrated through targeted re-contacting of a FG subcohort with a registry-based diagnosis of AD or MCD that in our recall setting cognitive testing can be performed through on-site clinical examination, a customized web-based tool, or remotely through phone interview, and that measures of episodic memory, executive function and verbal fluency correlate across all three platforms. We further showed a high level of overlap between FG registry data and hospital records and further validated the diagnosis AD versus MCD by determining blood-based AD biomarkers from freshly acquired blood samples.

Enabling recall of biobank participants for clinical follow-up studies is a new frontier for biobank research and considered as of substantial potential across academic and industry stakeholders. In fact, it has been suggested that the “bottom-up” targeted recruitment of individuals with distinct systematically-acquired biomolecular findings is likely to substantially accelerate the speed to validate the relevance of human genetic variation for disease^21^, or to generate functional insights on genetic discoveries. A few studies have given a glimpse on the potential of this approach. For instance, an earlier study in Finland in relatives of rare-variant carriers identified through population-level sequencing could substantiate the role of *SLC30A8* in insulin secretion and a Finn-enriched loss-of-function variant in that gene as protective for diabetes^22^. Other examples include recall studies in the Estonian Biobank or East London Genes and Health that guided medical interventions^23^ or informed transition of an early-stage rare disease therapeutic program towards the clinics^24^. Recall studies from population biobanks also hold considerable promise to improve clinical development of precision medicine drugs. Currently, a large fraction of interventional studies fail projected enrollment timelines, a challenge that could potentially be overcome through targeted outreach towards distinct, well-characterized subsets of population biobank participants with the highest projected probability to qualify for and benefit from inclusion into a clinical trial^25^. Despite such promises, numerous scientific, operational, and ethical challenges exist to firmly integrate biobank recall studies into the mainstream research, some of which impacted also to our study.

The more than 11,000 FG participants with a probable diagnosis AD at start of our study represent about 8% of prevalent dementia and AD cases in Finland^20^. As a research cohort, these individuals already contributed to improve the understanding of the genetic etiology of AD^26,27^. Utilizing research data from this cohort for a clinical recall study required thorough collaboration between the study investigators and BEF over a period of nearly two years from first contact to study completion. Specifically, a process needed to be developed that could in principle serve as a template for any future sample or clinical recall study from FG and the Finnish biobanks. Also, in accordance with the Finnish legislation, potential privacy concerns were mitigated by conducting all analyses in a secure compute environment, establishing recontact only through eligible biobank personnel, informing FG participants about their right to opt-out of continued use of their personal data for secondary use in research, and returning all results generated as part of a recall study to the respective biobank and FG for long-term storage and eventual public release.

While the sample size of our pilot recall study is too small for extrapolations to the entire FG population, it still speaks to the strength of the Finnish biobank system that for 93% (140/150) of the consented and targeted FG participants contact details were available, that 78% (110/140) could be reached through mail and recruitment calls within a period of less than three weeks, and that 30% (43/140) of patients and caregivers were in principle willing to enroll in the study. With 19% of contacted participants completing the study protocol, the final participation rate was in a similar range as that of an earlier questionnaire study where we had approached FG participants to provide cognitive, behavioral and lifestyle information via an online platform^28^, despite our current study targeting an aged and diseased population with a high vulnerability to a concomitantly ravaging SARS-Cov2 outbreak.

Registry data proved to be highly consistent with hospital records in our study population, indicating that clinical data captured in FG may in general be reliable and reproducible. As expected, however, registry data did not have the granularity to differentiate distinct courses of AD present among the trial participants. Another notable insight was that in only one of the 12 participants with ICD code F06.7 the mild cognitive disorder was attributable to AD. Researchers often consider mild cognitive impairment as a transitory phase from normal cognition to AD if no other obvious reasons for the cognitive deficits can be identified. We note that, while considered a proxy endpoint, F06.7 subsumes a broader disease spectrum than the mild cognitive impairment as defined in the National Institute on Aging-Alzheimer’s Association guidelines (30). In fact, our results propose that Finnish clinicians use this code to document the presence of cognitive impairment as part of medical conditions other than AD, reflected by eight different probable underlying conditions among the 12 individuals in the MCD subgroup. Our findings thus clearly advise against using F06.7 as a code with the aim of identifying early or pre-symptomatic AD patients from a biobank cohort.

For participants who completed the cognitive assessments via in-person, computerized and telephone-based testing, we observed a high correlation between MMSE and TELE. This is consistent with a previous study in Finnish patients with AD and healthy controls^17^. In comparison to MMSE and TELE, the CERAD-NB word list cut-off for immediate and delayed recall identified higher numbers of cognitively impaired individuals in both subgroups, emphasizing that studies like ours benefit from including more specific memory measures rather than simply relying on short screening instruments. Moderate to strong correlations between in-person (CERAD-NB) and computerized (cCOG) measures of immediate and delayed recall are in line with earlier studies comparing computerized/web-based versus in-person neuropsychological tests^29–32^. We also observed moderate to strong correlations between telephone-administered and in-person episodic memory measures. To our knowledge, our study is the first to use three learning trials of the 10-word list learning task included in the TICS-m. Thus, our telephone-based episodic memory measures closely corresponded to in-person word-list episodic memory measures. The lowest correlations between episodic memory measures were between TICS-m and cCOG word list measures: this could result from the fact these measures are based solely on verbal versus visual stimuli, respectively, whereas CERAD-NB word list includes both, visual and verbal stimuli. Based on the substantial inter-platform variability we observed in our study, future studies should be advised to assess episodic memory with more than a single test.

We observed significant associations between cCOG and in-person measures also for other cognitive domains. Processing speed, as assessed by TMT-A, showed a moderate association between in-person and cCOG assessment after exclusion of outliers. For TMT-B, measuring set-shifting, one of the core executive functions, showed a strong correlation between cCOG and in-person testing, which remained after accounting for processing speed and exclusion of outliers from TMT-A. These results are consistent with earlier findings^14^. However, given that a sizable fraction of participants was unable to complete the task, computer-based administration of TMT-A/B in an unfamiliar environment on site may be considered as too difficult for individuals with manifest AD or MCD. Notably though, we observed a strong association between in-person and telephone administered semantic fluency. Due to the fact that the 1-min animal naming was identical in both formats, this measure represented the strongest correlation among the measures of specific cognitive abilities. We are not aware of any other studies that have examined the association between in-person and telephone administered semantic fluency.

Despite the small size of our study, plasma levels of GFAP and pTau-181 were significantly different between the AD and MCD groups, which further supports the potential of these measures as diagnostic biomarkers for AD^33^. Conversely, no such differentiation was seen for Aβ42/40 ratio and NfL. Our results thus back recent findings that among the four plasma parameters pTau-181 might be the strongest differentiator of AD from non-AD patients^34^, which will need to be further validated at larger sample sizes. Larger studies will also be needed to thoroughly compare blood-based AD biomarker levels to cognitive performance. Notably, however, already in our current study we observed negative associations of TELE total score with GFAP, NfL, and pTau-181 levels, yet not with Aβ42/40 ratio. According to the expectations, there was a trend towards more *APOE* Σ4-carriers in the AD group than in the MCD group, with the prevalence of Σ4-carriers in the MCD group closely matching to the prevalence of over 30% in the Finnish population^35^.

Our study has several limitations: first, we invited participants from just a single of the nine Finnish biobanks contributing to FG and focused recruitment on a relatively uniform cohort in geographic proximity to the single study center. Follow-up studies that aim for a larger and more diverse study population will certainly face additional complexities, not the least with regards to harmonizing re-contacting, consenting, and contracting activities between multiple parties. It can be expected that the establishment of a centralized coordinating unit, such as the Finnish Biobank Cooperative FinBB, will facilitate the navigation through complex administrative processes that have not yet been customized for clinical recall activities in Finland and in our case required an extended period of planning. Second, according to FG records all individuals in our target population had already been diagnosed with either AD or MCD as a reflection of cognitive deficiencies. One of the greatest opportunities of biobank studies is to facilitate contact to individuals early in their disease or at risk, but not yet manifest disease, for instance because they carry genetic variants that without intervention reduce lifespan^8,36,37^. In fact, enrolling sufficient “trial-ready” minimally symptomatic AD patients into interventional trials often takes several years and may contribute substantially to the costs of clinical programs^38^. Third, the collection of on-site neuropsychological data was done before the TELE/TICS-m test in our protocol. This could cause systematic error in our results due to learning effects even when we tried to mitigate these by leaving a 1–2-week interval between the onsite visit and the remote assessment. Future studies should consider utilizing randomization of the participants to either complete the in-clinic visit or online/telephone data collection first. Fourth, our study protocol did not encompass a detailed re-evaluation of diagnoses at the time of the on-site visit. This was partly compensated by reviewing the participants’ medical records for possible disease progression data or misclassified diagnoses up to the time the study was conducted. It can thus not be excluded that participants in the MCD group may recently have developed AD or other neurodegenerative disease, but this had not yet been recorded in registry data or available hospital records. Finally, one could argue that with only 27 participants (19% of the original target population) our study might be at risk of selection bias, potentially altering the generalizability of our results. We consider this as unlikely since we did not observe obvious differences in basic demographic factors, either between the studied subcohorts, not the overall FG and wider Finnish population in this age group (https://risteys.finregistry.fi). Observed comorbidities closely resemble the profile of comorbid conditions in AD described in the literature^39^. The incidence of the two most frequent comorbidities in our study, hypertension and dyslipidemia, are also in line with the epidemiological data of these conditions in Finland^40^. We expect that future clinical recall studies from FG may yield higher enrollment rates by targeting a younger, healthier, and potentially more representative study population over a more extended enrollment period and by returning information that can lead to impactful health and lifestyle changes.

While FG participants are in principle consented for return of their results, studies in pre- and early-symptomatic individuals will need to carefully walk the fine line between providing useful information to improve a participant’s health or lifestyle and disclosing potentially unwanted and uncertain research information^41^. The correspondence between measures of cognitive function across different formats used in our study was generally good, we observed substantial variability of cognitive performance among the small number of individuals recalled for our study. This at least in part reflects that a few study participants were substantially further progressed in their disease than others. Further stratification based on available FG data may make future cohorts more homogenous. Also, cognitive function information acquired by phone in general tended to correlate better to in-person tests than information obtained via cCOG in our cohort; this may be due to our sample of individuals with AD having more problems with self-guided computerized tool versus using the phone. Finally, we note that with its small sample size our pilot study was not designed to detect robust statistical differences between measures, so results should be evaluated with caution.

In summary, our study introduces a recall framework to launch clinical studies from FG and the Finnish biobanks. While laborious to operationalize, the opportunity to recontact FG participants based on distinct genetic or phenotypic parameters opens the door for a broad spectrum of scientific activities, ranging from the validation of genetic findings in targeted follow-up experiments to delivering new and emerging precision treatments to individuals with the highest medical needs.

### Supplementary Information

Supplementary Table S1.

## Data Availability

The data that support the findings of this study are available in the FinnGen sandbox environment, but restrictions apply to the availability of these data, which were used under license for the current study, and so are not publicly available. Data are however available in the FinnGen sandbox upon reasonable request and with permission of FinnGen.
