# Supplementary material for: A FinnGen pilot clinical recall study for Alzheimer’s disease
Source: Sci Rep. 2023 Aug 3;13:12641. doi: 10.1038/s41598-023-39835-7 (PMC10400697; doi:10.1038/s41598-023-39835-7)
Supplement: Supplementary file 1 — Supplementary Table S1. [file 41598_2023_39835_MOESM1_ESM.pdf]

A FinnGen pilot clinical recall study for Alzheimer's disease

Valtteri Julkunen[1,2,3,#], Claudia Schwarz[3,4], Juho Kalapudas[1], Merja Hallikainen[1], Aino-Kaisa Piironen[5], Arto Mannermaa[5], Hanna Kujala[5], Timo Laitinen[5], Veli-Matti Kosma[5], Teemu I. Paajanen[6], Reetta Kälviäinen[1], Mikko Hiltunen[1], Sanna-Kaisa Herukka[1], Sari Kärkkäinen[1], Tarja Kokkola[1], Mia Urjansson[3], FinnGen, Markus Perola[7], Aarno Palotie[3,8,9,], Eero Vuoksimaa[3,*,#], Heiko Runz[10,*,#]

[1]*Institute of Clinical Medicine/Neurology, University of Eastern Finland, Kuopio, Finland*

[2]*Department of Neurology, Neurocenter, Kuopio University Hospital, Kuopio, Finland*

[3]*Institute for Molecular Medicine Finland (FIMM), HiLIFE, University of Helsinki, Finland*

[4]*Department of Neurology, University Medicine Greifswald, Greifswald, Germany*

[5]*Biobank of Eastern Finland, Kuopio, Finland*

[6]*Work Ability and Working Careers, Finnish Institute of Occupational Health, Helsinki, Finland*

[7]*Finnish Institute for Health and Welfare (THL), Helsinki, Finland*

[8]*Analytic and Translational Genetics Unit, Department of Medicine, Department of Neurology and Department of Psychiatry Massachusetts General Hospital, Boston, MA, USA*

[9]*The Stanley Center for Psychiatric Research and Program in Medical and Population Genetics, The Broad Institute of MIT and Harvard, Cambridge, MA, USA*

[10]*Translational Sciences, Biogen, Cambridge, MA, USA*

[*] equal contribution

[#] Correspondence: valtteri.julkunen@uef.fi, eero.vuoksimaa@helsinki.fi

or heiko.runz@gmail.com

Supplementary Table S1. Characteristics of the study cohort

	AD (n=15)	MCD (n=12)	p-value
**Education [n]**			1.000
less than high-school	6	5	
high-school and above	9	7	
**Smoking [n]**			0.400
once/week or more often	-	1	
former smoker	6	6	
never	9	5	
**Alcohol consumption [n]**			0.137
never	12	8	
once/month	2	-	
2-4x/month	-	3	
2-3x/week	1	1	
**Recurring Comorbidities* [n]**			
Hypertension	10	6	0.630
Dyslipidemia	7	8	0.516
Type 2 diabetes	<5	6	0.397
Depression	<5	5	0.681
At least 2 comorbidities*	15	12	1.000

All values are presented as number of subjects [n]. Statistical testing for significant differences across the groups was done using a chi-square test. The significance level was set at $\alpha = 0.05$.

*Additional comorbidities documented in individual study participants include cardiac diseases (coronary heart disease, atrial fibrillation, heart failure, arrhythmias, valvular diseases), different types of cancer (breast, prostate, skin), ophthalmologic diseases (cataract, glaucoma), musculoskeletal diseases (spinal stenosis, arthrosis, osteoporosis), neurological diseases (migraine, epilepsy, neuropathic pain syndromes, transient ischemic attack and cerebral infarction, Parkinson's disease), hypothyroidism, rheumatoid arthritis, sleep apnea, asthma and chronic obstructive pulmonary disease, prostate hyperplasia, atrophic gastritis, fibromyalgia, sleeping disorders.

AD, Alzheimer's disease; MCD, mild cognitive disorder.
